# Supplementary material for: Performance of microbiological tests for tuberculosis diagnostic according to the type of respiratory specimen: A 10-year retrospective study
Source: Front Cell Infect Microbiol. 2023 Mar 2;13:1131241. doi: 10.3389/fcimb.2023.1131241 (PMC10017756; doi:10.3389/fcimb.2023.1131241)
Supplement: Supplementary file 1 [file Table_1.docx]

# Supplementary material

**Performance of microbiological tests for tuberculosis diagnostic according to the type of respiratory specimen: a 10-year retrospective study**

Short title:

**Diagnostic tests for pulmonary tuberculosis**

## Table S1. P-values for Table 1, performance of smear microscopy according of the type of specimen.

| **MICROSCOPY** | **Sample1** | **Sample2** | **Diff_1m2** | **p.val** | **adj.p.val** | **sig.code** |
| --- | --- | --- | --- | --- | --- | --- |
| ACCURACY | All specimens | Sputum | 0.01004816 | 6.31016E-07 | 6.3102E-06 | *** |
| ACCURACY | All specimens | Induced sputum | 0.01129141 | 0.00118018 | 0.0118018 | * |
| ACCURACY | All specimens | Bronchial aspirate | -0.0079934 | 1.04282E-05 | 0.00010428 | *** |
| ACCURACY | All specimens | BAL | -0.0088853 | 0.000184048 | 0.00184048 | ** |
| ACCURACY | Sputum | Induced sputum | 0.00124324 | 0.823873939 | 1 | n.s. |
| ACCURACY | Sputum | Bronchial aspirate | -0.0180416 | 1.83229E-14 | 1.8323E-13 | *** |
| ACCURACY | Sputum | BAL | -0.0189335 | 1.73634E-10 | 1.7363E-09 | *** |
| ACCURACY | Induced sputum | Bronchial aspirate | -0.0192848 | 5.08274E-09 | 5.0827E-08 | *** |
| ACCURACY | Induced sputum | BAL | -0.0201767 | 7.30806E-08 | 7.3081E-07 | *** |
| ACCURACY | Bronchial aspirate | BAL | -0.0008919 | 0.741491376 | 1 | n.s. |
| SENSITIVITY | All specimens | Sputum | -0.0821079 | 0.003383206 | 0.03383206 | * |
| SENSITIVITY | All specimens | Induced sputum | 0.16110489 | 0.004279429 | 0.04279429 | * |
| SENSITIVITY | All specimens | Bronchial aspirate | 0.16110489 | 0.000552365 | 0.00552365 | ** |
| SENSITIVITY | All specimens | BAL | 0.06126856 | 0.316540154 | 1 | n.s. |
| SENSITIVITY | Sputum | Induced sputum | 0.24321281 | 1.92189E-05 | 0.00019219 | *** |
| SENSITIVITY | Sputum | Bronchial aspirate | 0.24321281 | 4.0431E-07 | 4.04E-06 | *** |
| SENSITIVITY | Sputum | BAL | 0.14337647 | 0.014367647 | 0.14367647 | n.s. |
| SENSITIVITY | Induced sputum | Bronchial aspirate | 0 | 1 | 1 | n.s. |
| SENSITIVITY | Induced sputum | BAL | -0.0998363 | 0.218542283 | 1 | n.s. |
| SENSITIVITY | Bronchial aspirate | BAL | -0.0998363 | 0.168260529 | 1 | n.s. |
| SPECIFICITY | All specimens | Sputum | 0.00269265 | 0.013860132 | 0.13860132 | n.s. |
| SPECIFICITY | All specimens | Induced sputum | 0.00120607 | 0.592195013 | 1 | n.s. |
| SPECIFICITY | All specimens | Bronchial aspirate | -0.0014397 | 0.150681561 | 1 | n.s. |
| SPECIFICITY | All specimens | BAL | -0.0025871 | 0.042568282 | 0.42568282 | n.s. |
| SPECIFICITY | Sputum | Induced sputum | -0.0014866 | 0.595177457 | 1 | n.s. |
| SPECIFICITY | Sputum | Bronchial aspirate | -0.0041324 | 0.001430623 | 0.01430623 | * |
| SPECIFICITY | Sputum | BAL | -0.0052797 | 0.000839022 | 0.00839022 | ** |
| SPECIFICITY | Induced sputum | Bronchial aspirate | -0.0026458 | 0.173082897 | 1 | n.s. |
| SPECIFICITY | Induced sputum | BAL | -0.0037931 | 0.053324693 | 0.53324693 | n.s. |
| SPECIFICITY | Bronchial aspirate | BAL | -0.0011473 | 0.408352919 | 1 | n.s. |
| PREVALENCE | All specimens | Sputum | -0.0269285 | 1.84099E-25 | 1.841E-24 | *** |
| PREVALENCE | All specimens | Induced sputum | -0.0068638 | 0.11419982 | 1 | n.s. |
| PREVALENCE | All specimens | Bronchial aspirate | 0.01956778 | 3.18425E-19 | 3.1843E-18 | *** |
| PREVALENCE | All specimens | BAL | 0.01606347 | 4.01497E-08 | 4.015E-07 | *** |
| PREVALENCE | Sputum | Induced sputum | 0.02006467 | 0.000451444 | 0.00451444 | ** |
| PREVALENCE | Sputum | Bronchial aspirate | 0.04649626 | 1.90752E-54 | 1.9075E-53 | *** |
| PREVALENCE | Sputum | BAL | 0.04299196 | 8.81979E-28 | 8.8198E-27 | *** |
| PREVALENCE | Induced sputum | Bronchial aspirate | 0.0264316 | 7.06582E-14 | 7.0658E-13 | *** |
| PREVALENCE | Induced sputum | BAL | 0.02292729 | 9.71951E-08 | 9.7195E-07 | *** |
| PREVALENCE | Bronchial aspirate | BAL | -0.0035043 | 0.166135822 | 1 | n.s. |
| NPV | All specimens | Sputum | 0.00845522 | 3.45875E-06 | 3.4587E-05 | *** |
| NPV | All specimens | Induced sputum | 0.03207957 | 6.34282E-24 | 6.3428E-23 | *** |
| NPV | All specimens | Bronchial aspirate | -0.0070156 | 1.09507E-05 | 0.00010951 | *** |
| NPV | All specimens | BAL | -0.0067415 | 0.001431937 | 0.01431937 | * |
| NPV | Sputum | Induced sputum | 0.02362435 | 2.45115E-08 | 2.4512E-07 | *** |
| NPV | Sputum | Bronchial aspirate | -0.0154709 | 1.38418E-13 | 1.3842E-12 | *** |
| NPV | Sputum | BAL | -0.0151967 | 1.30343E-08 | 1.3034E-07 | *** |
| NPV | Induced sputum | Bronchial aspirate | -0.0390952 | 3.82866E-33 | 3.8287E-32 | *** |
| NPV | Induced sputum | BAL | -0.038821 | 1.88248E-22 | 1.8825E-21 | *** |
| NPV | Bronchial aspirate | BAL | 0.00027418 | 0.955959595 | 1 | n.s. |
| PPV | All specimens | Sputum | -0.0580184 | 0.036317752 | 0.36317752 | n.s. |
| PPV | All specimens | Induced sputum | 0.07284629 | 0.329999789 | 1 | n.s. |
| PPV | All specimens | Bronchial aspirate | 0.19369013 | 0.000173017 | 0.00173017 | ** |
| PPV | All specimens | BAL | 0.02988174 | 0.731065417 | 1 | n.s. |
| PPV | Sputum | Induced sputum | 0.13086472 | 0.044715742 | 0.44715742 | n.s. |
| PPV | Sputum | Bronchial aspirate | 0.25170856 | 5.44063E-07 | 5.4406E-06 | *** |
| PPV | Sputum | BAL | 0.08790016 | 0.158902886 | 1 | n.s. |
| PPV | Induced sputum | Bronchial aspirate | 0.12084384 | 0.224805481 | 1 | n.s. |
| PPV | Induced sputum | BAL | -0.0429646 | 0.784581394 | 1 | n.s. |
| PPV | Bronchial aspirate | BAL | -0.1638084 | 0.067009051 | 0.67009051 | n.s. |

## Table S2. P-values for Table 2, performance of PCR according of the type of specimen.

| **PCR** | **Sample1** | **Sample2** | **Diff_1m2** | **p.val** | **adj.p.val** | **sig.code** |
| --- | --- | --- | --- | --- | --- | --- |
| ACCURACY | All specimens | Sputum | 0.00287789 | 0.304418176 | 1 | n.s. |
| ACCURACY | All specimens | Induced sputum | 0.01147752 | 0.019512972 | 0.19512972 | n.s. |
| ACCURACY | All specimens | Bronchial aspirate | -0.0040849 | 0.123765813 | 1 | n.s. |
| ACCURACY | All specimens | BAL | -0.0034643 | 0.283404464 | 1 | n.s. |
| ACCURACY | Sputum | Induced sputum | 0.00859963 | 0.167946429 | 1 | n.s. |
| ACCURACY | Sputum | Bronchial aspirate | -0.0069627 | 0.030595877 | 0.30595877 | n.s. |
| ACCURACY | Sputum | BAL | -0.0063422 | 0.092258909 | 0.92258909 | n.s. |
| ACCURACY | Induced sputum | Bronchial aspirate | -0.0155624 | 0.001625285 | 0.01625285 | * |
| ACCURACY | Induced sputum | BAL | -0.0149418 | 0.007047431 | 0.07047431 | n.s. |
| ACCURACY | Bronchial aspirate | BAL | 0.00062059 | 0.975120507 | 1 | n.s. |
| SENSITIVITY | All specimens | Sputum | -0.0232511 | 0.562771431 | 1 | n.s. |
| SENSITIVITY | All specimens | Induced sputum | 0.15514784 | 0.033580028 | 0.33580028 | n.s. |
| SENSITIVITY | All specimens | Bronchial aspirate | -0.0387297 | 0.467749275 | 1 | n.s. |
| SENSITIVITY | All specimens | BAL | 0.03874573 | 0.630382277 | 1 | n.s. |
| SENSITIVITY | Sputum | Induced sputum | 0.1783989 | 0.017069317 | 0.17069317 | n.s. |
| SENSITIVITY | Sputum | Bronchial aspirate | -0.0154787 | 0.863947971 | 1 | n.s. |
| SENSITIVITY | Sputum | BAL | 0.06199678 | 0.402691103 | 1 | n.s. |
| SENSITIVITY | Induced sputum | Bronchial aspirate | -0.1938776 | 0.020902399 | 0.20902399 | n.s. |
| SENSITIVITY | Induced sputum | BAL | -0.1164021 | 0.308470102 | 1 | n.s. |
| SENSITIVITY | Bronchial aspirate | BAL | 0.07747543 | 0.343242299 | 1 | n.s. |
| SPECIFICITY | All specimens | Sputum | -0.0001495 | 1 | 1 | n.s. |
| SPECIFICITY | All specimens | Induced sputum | -0.0001495 | 1 | 1 | n.s. |
| SPECIFICITY | All specimens | Bronchial aspirate | -0.0001495 | 1 | 1 | n.s. |
| SPECIFICITY | All specimens | BAL | -0.0001495 | 1 | 1 | n.s. |
| SPECIFICITY | Sputum | Induced sputum | 0 | NA | NA |  |
| SPECIFICITY | Sputum | Bronchial aspirate | 0 | NA | NA |  |
| SPECIFICITY | Sputum | BAL | 0 | NA | NA |  |
| SPECIFICITY | Induced sputum | Bronchial aspirate | 0 | NA | NA |  |
| SPECIFICITY | Induced sputum | BAL | 0 | NA | NA |  |
| SPECIFICITY | Bronchial aspirate | BAL | 0 | NA | NA |  |
| PREVALENCE | All specimens | Sputum | -0.0242457 | 1.85046E-05 | 0.00018505 | *** |
| PREVALENCE | All specimens | Induced sputum | -0.012415 | 0.24121711 | 1 | n.s. |
| PREVALENCE | All specimens | Bronchial aspirate | 0.01343573 | 0.015178942 | 0.15178942 | n.s. |
| PREVALENCE | All specimens | BAL | 0.0229057 | 0.000252936 | 0.00252936 | ** |
| PREVALENCE | Sputum | Induced sputum | 0.01183074 | 0.372502519 | 1 | n.s. |
| PREVALENCE | Sputum | Bronchial aspirate | 0.03768143 | 1.03816E-07 | 1.0382E-06 | *** |
| PREVALENCE | Sputum | BAL | 0.04715139 | 1.47378E-09 | 1.4738E-08 | *** |
| PREVALENCE | Induced sputum | Bronchial aspirate | 0.02585069 | 0.011433057 | 0.11433057 | n.s. |
| PREVALENCE | Induced sputum | BAL | 0.03532066 | 0.000420219 | 0.00420219 | ** |
| PREVALENCE | Bronchial aspirate | BAL | 0.00946997 | 0.157733443 | 1 | n.s. |
| NPV | All specimens | Sputum | 0.0355276 | 1.72723E-11 | 1.7272E-10 | *** |
| NPV | All specimens | Induced sputum | -0.0212302 | 0.016901135 | 0.16901135 | n.s. |
| NPV | All specimens | Bronchial aspirate | -0.0448089 | 3.59424E-23 | 3.5942E-22 | *** |
| NPV | All specimens | BAL | -0.0369566 | 1.0787E-11 | 1.0787E-10 | *** |
| NPV | Sputum | Induced sputum | -0.0567578 | 9.29799E-07 | 9.298E-06 | *** |
| NPV | Sputum | Bronchial aspirate | -0.0803365 | 8.28082E-41 | 8.2808E-40 | *** |
| NPV | Sputum | BAL | -0.0724842 | 1.81678E-23 | 1.8168E-22 | *** |
| NPV | Induced sputum | Bronchial aspirate | -0.0235787 | 4.52576E-11 | 4.5258E-10 | *** |
| NPV | Induced sputum | BAL | -0.0157264 | 0.006062577 | 0.06062577 | n.s. |
| NPV | Bronchial aspirate | BAL | 0.00785225 | 0.00031041 | 0.0031041 | ** |
| PPV | All specimens | Sputum | -0.0031153 | 1 | 1 | n.s. |
| PPV | All specimens | Induced sputum | -0.0031153 | 1 | 1 | n.s. |
| PPV | All specimens | Bronchial aspirate | -0.0031153 | 1 | 1 | n.s. |
| PPV | All specimens | BAL | -0.0031153 | 1 | 1 | n.s. |
| PPV | Sputum | Induced sputum | 0 | NA | NA |  |
| PPV | Sputum | Bronchial aspirate | 0 | NA | NA |  |
| PPV | Sputum | BAL | 0 | NA | NA |  |
| PPV | Induced sputum | Bronchial aspirate | 0 | NA | NA |  |
| PPV | Induced sputum | BAL | 0 | NA | NA |  |
| PPV | Bronchial aspirate | BAL | 0 | NA | NA |  |

## Table S3. P-values for Table 3, performance of culture according of the type of specimen.

| **CULTURE** | **Sample1** | **Sample2** | **Diff_1m2** | **p.val** | **adj.p.val** | **sig.code** |
| --- | --- | --- | --- | --- | --- | --- |
| ACCURACY | All specimens | Sputum | 8.0593E-06 | 1 | 1 | n.s. |
| ACCURACY | All specimens | Induced sputum | 0.00042837 | 0.70389402 | 1 | n.s. |
| ACCURACY | All specimens | Bronchial aspirate | 0.00012296 | 0.8740244 | 1 | n.s. |
| ACCURACY | All specimens | BAL | -0.0004578 | 0.30008201 | 1 | n.s. |
| ACCURACY | Sputum | Induced sputum | 0.00042031 | 0.80051808 | 1 | n.s. |
| ACCURACY | Sputum | Bronchial aspirate | 0.0001149 | 1 | 1 | n.s. |
| ACCURACY | Sputum | BAL | -0.0004658 | 0.34969855 | 1 | n.s. |
| ACCURACY | Induced sputum | Bronchial aspirate | -0.0003054 | 0.96569159 | 1 | n.s. |
| ACCURACY | Induced sputum | BAL | -0.0008861 | 0.20689472 | 1 | n.s. |
| ACCURACY | Bronchial aspirate | BAL | -0.0005807 | 0.24579232 | 1 | n.s. |
| SENSITIVITY | All specimens | Sputum | -0.005159 | 0.50725204 | 1 | n.s. |
| SENSITIVITY | All specimens | Induced sputum | 0.00713619 | 0.88895376 | 1 | n.s. |
| SENSITIVITY | All specimens | Bronchial aspirate | 0.02298934 | 0.09347319 | 0.93473193 | n.s. |
| SENSITIVITY | All specimens | BAL | -0.0124717 | 0.57625897 | 1 | n.s. |
| SENSITIVITY | Sputum | Induced sputum | 0.01229523 | 0.53019702 | 1 | n.s. |
| SENSITIVITY | Sputum | Bronchial aspirate | 0.02814838 | 0.02728883 | 0.27288835 | n.s. |
| SENSITIVITY | Sputum | BAL | -0.0073126 | 0.91364702 | 1 | n.s. |
| SENSITIVITY | Induced sputum | Bronchial aspirate | 0.01585315 | 0.7334086 | 1 | n.s. |
| SENSITIVITY | Induced sputum | BAL | -0.0196078 | 0.5232443 | 1 | n.s. |
| SENSITIVITY | Bronchial aspirate | BAL | -0.035461 | 0.17273125 | 1 | n.s. |
| SPECIFICITY | All specimens | Sputum | 0 | NA | NA |  |
| SPECIFICITY | All specimens | Induced sputum | 0 | NA | NA |  |
| SPECIFICITY | All specimens | Bronchial aspirate | 0 | NA | NA |  |
| SPECIFICITY | All specimens | BAL | 0 | NA | NA |  |
| SPECIFICITY | Sputum | Induced sputum | 0 | NA | NA |  |
| SPECIFICITY | Sputum | Bronchial aspirate | 0 | NA | NA |  |
| SPECIFICITY | Sputum | BAL | 0 | NA | NA |  |
| SPECIFICITY | Induced sputum | Bronchial aspirate | 0 | NA | NA |  |
| SPECIFICITY | Induced sputum | BAL | 0 | NA | NA |  |
| SPECIFICITY | Bronchial aspirate | BAL | 0 | NA | NA |  |
| PREVALENCE | All specimens | Sputum | -0.0269968 | 1.3127E-25 | 1.3127E-24 | *** |
| PREVALENCE | All specimens | Induced sputum | -0.0106277 | 0.01526368 | 0.15263681 | n.s. |
| PREVALENCE | All specimens | Bronchial aspirate | 0.02032781 | 2.1937E-20 | 2.1937E-19 | *** |
| PREVALENCE | All specimens | BAL | 0.01659922 | 1.7986E-08 | 1.7986E-07 | *** |
| PREVALENCE | Sputum | Induced sputum | 0.01636916 | 0.00509466 | 0.05094659 | n.s. |
| PREVALENCE | Sputum | Bronchial aspirate | 0.04732464 | 3.3813E-56 | 3.3813E-55 | *** |
| PREVALENCE | Sputum | BAL | 0.04359605 | 2.4327E-28 | 2.4327E-27 | *** |
| PREVALENCE | Induced sputum | Bronchial aspirate | 0.03095548 | 1.0264E-17 | 1.0264E-16 | *** |
| PREVALENCE | Induced sputum | BAL | 0.02722689 | 7.7062E-10 | 7.7062E-09 | *** |
| PREVALENCE | Bronchial aspirate | BAL | -0.0037286 | 0.13952978 | 1 | n.s. |
| NPV | All specimens | Sputum | 2.2288E-05 | 1 | 1 | n.s. |
| NPV | All specimens | Induced sputum | -0.000475 | 0.63719344 | 1 | n.s. |
| NPV | All specimens | Bronchial aspirate | 0.00011506 | 0.90389031 | 1 | n.s. |
| NPV | All specimens | BAL | -0.000475 | 0.29342131 | 1 | n.s. |
| NPV | Sputum | Induced sputum | -0.0004973 | 0.67239509 | 1 | n.s. |
| NPV | Sputum | Bronchial aspirate | 9.2775E-05 | 1 | 1 | n.s. |
| NPV | Sputum | BAL | -0.0004973 | 0.33110675 | 1 | n.s. |
| NPV | Induced sputum | Bronchial aspirate | 0.00059004 | 0.56760236 | 1 | n.s. |
| NPV | Induced sputum | BAL | 0 | NA | NA |  |
| NPV | Bronchial aspirate | BAL | -0.00059 | 0.24737688 | 1 | n.s. |
| PPV | All specimens | Sputum | 0 | NA | NA |  |
| PPV | All specimens | Induced sputum | 0 | NA | NA |  |
| PPV | All specimens | Bronchial aspirate | 0 | NA | NA |  |
| PPV | All specimens | BAL | 0 | NA | NA |  |
| PPV | Sputum | Induced sputum | 0 | NA | NA |  |
| PPV | Sputum | Bronchial aspirate | 0 | NA | NA |  |
| PPV | Sputum | BAL | 0 | NA | NA |  |
| PPV | Induced sputum | Bronchial aspirate | 0 | NA | NA |  |
| PPV | Induced sputum | BAL | 0 | NA | NA |  |
| PPV | Bronchial aspirate | BAL | 0 | NA | NA |  |

## Table S4. P-values for Table 4, performance of smear microscopy according of the type of specimen.

| **Table S4. P-values for table 4, performance of smear microscopy according of the type of specimen.** | | | | | | |
| --- | --- | --- | --- | --- | --- | --- |
| **Pairwise Comparison Direct Exam** | **Sample1** | **Sample2** | **Diff_1m2** | **p.val** | **adj.p.val** | **sig.code** |
| SENSITIVITY | Sputum | Induced sputum | 0.15384615 | 0.58674615 | 1 | n.s. |
| SENSITIVITY | Sputum | Bronchial aspirate | -0.1538462 | 0.64159157 | 1 | n.s. |
| SENSITIVITY | Sputum | BAL | 0.25 | 0.4496918 | 1 | n.s. |
| SENSITIVITY | Induced sputum | Bronchial aspirate | 0 | 1 | 1 | n.s. |
| SENSITIVITY | Induced sputum | BAL | -0.3333333 | 1 | 1 | n.s. |
| SENSITIVITY | Bronchial aspirate | BAL | 0.16666667 | 0.10322732 | 0.61936393 | n.s. |
| NPV | Sputum | Induced sputum | 0.00507604 | 0.61977665 | 1 | n.s. |
| NPV | Sputum | Bronchial aspirate | -0.0058305 | 0.68707568 | 1 | n.s. |
| NPV | Sputum | BAL | 0.0070922 | 0.48184948 | 1 | n.s. |
| NPV | Induced sputum | Bronchial aspirate | 0 | 1 | 1 | n.s. |
| NPV | Induced sputum | BAL | -0.0188537 | 1 | 1 | n.s. |
| NPV | Bronchial aspirate | BAL | 0.0019746 | 0.14625681 | 0.87754084 | n.s. |

## Table S5. P-values for Table 5, sensitivity of PCR to predict tuberculosis according to the type of specimen using a 72 72-hours pairing window in the same patient..

| **Table S5. P.values for table 5, sensitivity of PCR to predict tuberculosis according to the type of specimen using a 72-hours pairing window in the same patient..** | | | | | | |
| --- | --- | --- | --- | --- | --- | --- |
| **PAIRWISE COMPARISON PCR** | **Sample1** | **Sample2** | **Diff_1m2** | **p.val** | **adj.p.val** | **sig.code** |
| SENSITIVITY | Sputum | Induced sputum | 0 | 1 | 1 | n.s. |
| SENSITIVITY | Sputum | Bronchial aspirate | -0.6153846 | 0.00293535 | 0.01761212 | * |
| SENSITIVITY | Sputum | BAL | -0.4 | 0.4291953 | 1 | n.s. |
| SENSITIVITY | Induced sputum | Bronchial aspirate | 0.14285714 | 1 | 1 | n.s. |
| SENSITIVITY | Induced sputum | BAL | 0.4 | 0.4291953 | 1 | n.s. |
| SENSITIVITY | Bronchial aspirate | BAL | 0.41025641 | 5.551E-05 | 0.00033306 | *** |
| NPV | Sputum | Induced sputum | 0 | 1 | 1 | n.s. |
| NPV | Sputum | Bronchial aspirate | -0.0473373 | 0.01482072 | 0.08892431 | n.s. |
| NPV | Sputum | BAL | -0.0149254 | 0.48447295 | 1 | n.s. |
| NPV | Induced sputum | Bronchial aspirate | 0.03024194 | 1 | 1 | n.s. |
| NPV | Induced sputum | BAL | 0.0625 | 0.50109265 | 1 | n.s. |
| NPV | Bronchial aspirate | BAL | 0.01379293 | 0.00043168 | 0.00259007 | ** |

## Table S6. P.values for table 1, sensitivity of culture to predict tuberculosis according to the type of specimen using a 72-hour pairing window within the same patient.

| **Table S4. P.values for table 6, sensitivity of culture to predict tuberculosis according to the type of specimen using a 72 72-hours pairing window within the same patient..** | | | | | | |
| --- | --- | --- | --- | --- | --- | --- |
| **PAIRWISE CULTURE** | **Sample1** | **Sample2** | **Diff_1m2** | **p.val** | **adj.p.val** | **sig.code** |
| SENSITIVITY | Sputum | Induced sputum | 0.05714286 | 0.75114104 | 1 | n.s. |
| SENSITIVITY | Sputum | Bronchial aspirate | -0.2903226 | 0.01767207 | 0.10603241 | n.s. |
| SENSITIVITY | Sputum | BAL | 0.10526316 | 0.65640773 | 1 | n.s. |
| SENSITIVITY | Induced sputum | Bronchial aspirate | -0.1538462 | 0.64159157 | 1 | n.s. |
| SENSITIVITY | Induced sputum | BAL | 0.30769231 | 0.22653276 | 1 | n.s. |
| SENSITIVITY | Bronchial aspirate | BAL | 0.33333333 | 1.0772E-08 | 6.4633E-08 | *** |
| NPV | Induced sputum | 0.00526184 | 0.77818701 | 1 | n.s. |  |
| NPV | Bronchial aspirate | -0.0270178 | 0.04176735 | 0.25060407 | n.s. |  |
| NPV | BAL | 0.00729847 | 0.68809585 | 1 | n.s. |  |
| NPV | Bronchial aspirate | -0.0406619 | 0.71342494 | 1 | n.s. |  |
| NPV | BAL | 0.08074534 | 0.39206894 | 1 | n.s. |  |
| NPV | BAL | 0.00940163 | 3.2399E-07 | 1.944E-06 | *** |  |
| E=sputum (spontaneous expectoration), Induced sputum=Induced sputum, Bronchial aspirate=bronchial aspirate, BAL=Bronchoalveolar lavage. | | | | | | |

## Table S7. Performance of in-house Taqman PCR and Xpert for the diagnostic of pulmonary tuberculosis.

| **Supplementary table 2. Performance of in-house Taqman PCR and Xpert for the diagnostic of pulmonary tuberculosis.** | | | | | | |
| --- | --- | --- | --- | --- | --- | --- |
|  | **Accuracy** | **Sensitivity** | **Specificity** | **Prevalence** | **PPV** | **NPV** |
| **In-house TaqMan PCR** | 0.991 (0.988, 0.994) 5493/5542 | 0.799  (0.743, 0.848) 191/239 | >0.999 (0.999, 1) 5302/5303 | 0.043 (0.038, 0.049) 239/5542 | 0.995 (0.971, 1) 191/192 | 0.991 (0.988, 0.993) 5302/5350 |
| **Xpert** | 0.975 (0.967, 0.981) 1917/1967 | 0.812 (0.760, 0.858) 212/261 | 0.999 (0.997, 1) 1705/1706 | 0.133 (0.118, 0.148) 261/1967 | 0.995 (0.974, 1) 212/213 | 0.972 (0.963, 0.979) 1705/1754 |

## Table S8. Kappa with 95% confidence intervals.

|  | Microscopy | PCR | Culture |
| --- | --- | --- | --- |
| Sputum vs Induced sputum | 0.73 (0.54,0.92) | 0.49 (-0.11,1) | 0.78 (0.66,0.9) |
| Sputum vs Bronchial aspirate | 0.79 (0.62,0.97) | 0.54 (0.26,0.81) | 0.66 (0.50,0.82) |
| Sputum vs BAL | 0.93 (0.80,1) | 0.74 (0.40,1) | 0.80 (0.65,0.96) |
| Induced sputum vs Bronchial aspirate | -0.02 (-0.04,0.01) | 0.68 (0.34,1) | 0.64 (0.37,0.90) |
| Induced sputum vs BAL | -0.02 (-0.05,0.01) | 0.72 (0.36,1) | 0.27 (-0.06,0.60) |
| Bronchial aspirate vs BAL | 0.72 (0.62,0.82) | 0.69 (0.56,1) | 0.75 (0.67,0.83) |

## Table S9. Contingency table for kappa: microscopy

|  | Induced sputum | |
| --- | --- | --- |
| Sputum | 1 | 0 |
| 1 | 10 | 5 |
| 0 | 2 | 364 |
|  | Bronchial aspirate | |
| Sputum | 1 | 0 |
| 1 | 10 | 1 |
| 0 | 4 | 332 |
|  | BAL | |
| Sputum | 1 | 0 |
| 1 | 7 | 1 |
| 0 | 0 | 278 |
|  | Bronchial aspirate | |
| Induced sputum | 1 | 0 |
| 1 | 0 | 1 |
| 0 | 1 | 54 |
|  | BAL | |
| Induced sputum | 1 | 0 |
| 1 | 0 | 1 |
| 0 | 1 | 50 |
|  | BAL | |
| Bronchial aspirate | 1 | 0 |
| 1 | 35 | 18 |
| 0 | 9 | 3502 |

## Table S10. Contingency table for kappa: PCR

|  | Induced sputum | |
| --- | --- | --- |
| Sputum | 1 | 0 |
| 1 | 1 | 150 |
| 0 | 129 | 1927 |
|  | Bronchial aspirate | |
| Sputum | 1 | 0 |
| 1 | 0 | 147 |
| 0 | 161 | 1888 |
|  | BAL | |
| Sputum | 1 | 0 |
| 1 | 0 | 149 |
| 0 | 132 | 1923 |
|  | Bronchial aspirate | |
| Induced sputum | 1 | 0 |
| 1 | 2 | 77 |
| 0 | 30 | 2144 |
|  | BAL | |
| Induced sputum | 1 | 0 |
| 1 | 3 | 2 |
| 0 | 0 | 30 |
|  | BAL | |
| Bronchial aspirate | 1 | 0 |
| 1 | 21 | 18 |
| 0 | 1141 | 1033 |

## Table S11. Contingency table for kappa: culture

|  | Induced sputum | |
| --- | --- | --- |
| Sputum | 1 | 0 |
| 1 | 23 | 7 |
| 0 | 5 | 368 |
|  | Bronchial aspirate | |
| Sputum | 1 | 0 |
| 1 | 16 | 3 |
| 0 | 12 | 318 |
|  | BAL | |
| Sputum | 1 | 0 |
| 1 | 13 | 4 |
| 0 | 2 | 268 |
|  | Bronchial aspirate | |
| Induced sputum | 1 | 0 |
| 1 | 7 | 2 |
| 0 | 4 | 43 |
|  | BAL | |
| Induced sputum | 1 | 0 |
| 1 | 3 | 7 |
| 0 | 3 | 39 |
|  | BAL | |
| Bronchial aspirate | 1 | 0 |
| 1 | 60 | 36 |
| 0 | 3 | 3471 |
